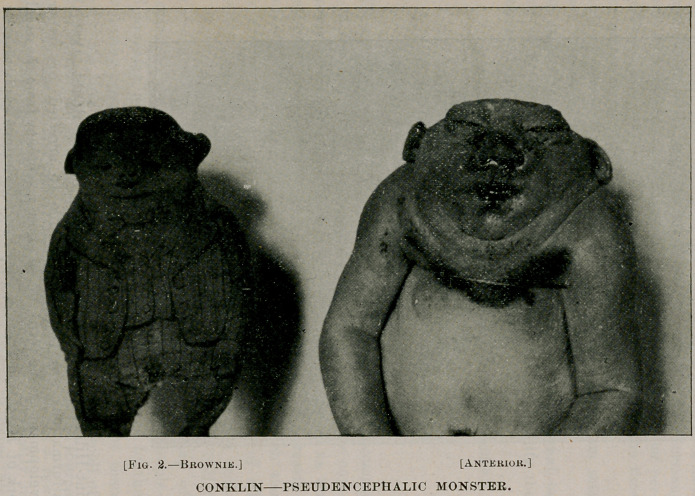# A Pseudencephalic Monster

**Published:** 1896-03

**Authors:** William L. Conklin

**Affiliations:** Rochester, N. Y.


### A PSEUDENCEPHALIC MONSTER.

By WILLIAM L. CONKLIN, M. D., Rochester, N. Y.

THE specimen which I present is not of special interest because of its extreme rarity, for it belongs to one of the more common forms of malformations. It is of interest, however, as an illustration of the fact that there is, at times, a striking resemblance between the malformed fetus and some object which the mother and her friends are firmly convinced was the source of all the trouble. This resemblance is often fancied rather than real, but it is, doubtless, largely responsible for the widespread belief among the laity that every deviation from the normal, in any of the varying degrees, from a strawberry on the arm to a double-headed monster, is due to some mental impression received by the mother during pregnancy. This is a less unfortunate error than the older one that these monsters were due to the potent spell of some evil spirit; but it is an error and one, too, which is the source of much needless anxiety and distress to many mothers, not a few of whom are intelligent in regard to other subjects. Evidently it is the duty of the physician to do all in his power to dispel this prevalent error. He must, however, first rid his own mind of any lingering doubt as to the "maternal impression" theory and be able to speak very positively in regard to the subject. There was a time when this theory had its advocates among intelligent physi-

1. Read at the twenty-eighth annual meeting of the Medical Association of Central New York, at Syracuse, October 15, 1895.

cians, and, if I mistake not, there are yet to be found those whose views in regard to the subject are not entirely settled.

The study of embryology has done much to clear up this somewhat obscure subject and has furnished a basis of facts for our present knowledge of teratology. The investigations of Goeffroy,

[Fig. 1.—POSTERIOR.]

CONKLIN—PSEUDENCEPHALIC MONSTER.

Saint-Hilaire and others have proven, beyond doubt, that these malformations are very largely due to arrested or imperfect development of the fetus, or, to quote a sentence from the excellent work of Hirst and Piersol, they are "deviations explicable by the application of the definite laws of development."

In the work just referred to, the following simple classification of human deformities is given:

I. Those produced by variations in growth, either excessive or arrested.

II. Those produced by defective union of component, embryonal parts.

III. Those produced by cleavage (either partial or complete) of the primary embryonal cell masses.

While there are some malformations which are the result of

[Fig. 2.—Brownie.] [Anterior.]

CONKLIN—PSEUDENCEPHALIC MONSTER.

more than one of these causes, and others which may be due, either wholly or in part, to pathological processes, *e. g.*, inflammatory changes, still the large majority may be placed in one of these classes. "Increased intracranial pressure, resulting in rupture of the early cerebral vesicle," is mentioned by Hirst and Piersol as a possible cause of anencephalus; but they observe that "the rudimentary condition often observed of the basal portions of the cranium and of the upper cervical vertebra, bears additional testimony to the influence of a condition of primary arrest of development."

The term anencephalic is often used in describing the various forms of malformation resulting from arrested brain development. Saint-Hilaire, however, applies it exclusively to cases in which there is no trace of brain tissue to be found, and uses the term pseudencephalic in describing cases in which the brain is replaced by a mass of connective and membranous tissue, blood-vessels and possibly traces of nerve tissue. The specimen presented belongs to the latter class, as there is such a conglomerate mass of tissue at the base of the skull. The occiput is wanting and the other cranial bones either absent or very imperfectly developed. This faulty development of both brain and skull, together with a similar defect in the cervical vertebræ, accounts for the peculiar physiognomy which has been aptly described by the term "frog-headed."

It is said that malformations of this class may be diagnosticated during pregnancy by the presence of hydramnios and of fetal movements which are unusual in character. In this case both of these conditions were present. The amount of liquor amnii was so large that a literal deluge followed the rupture of the membranes. Labor came on about six weeks before the expected time and was tedious in character. The head presented, but the absence of the occipital bone made an early diagnosis of the presentation decidedly difficult. A day or two after the confinement a sister of the patient, informing me that she had discovered the cause of all the trouble, produced a Palmer Cox brownie, which, it will be seen, has the grotesque features characteristic of that remarkable family. It had been left in the yard by the children and at an early period of her pregnancy the mother had stepped on it, and thinking it was a toad, for which animal she has a special abhorrence, she was much frightened. As the photograph will show, there is a striking resemblance between the brownie and the baby, and it is not strange, in view of the popular belief, that the former was held responsible for the peculiar physiognomy of the latter. These resemblances, whether fancied or real, furnish an argument in favor of the mental impression theory, which, in the minds of many of the laity, is still unanswerable. Let us hope, however, that the time is not far distant when this popular error shall give place to a better understanding of the subject and when mothers shall no longer be haunted with the fear of "marking the baby."

96 South Avenue.

## Figures and Tables

**Fig. 1. f1:**
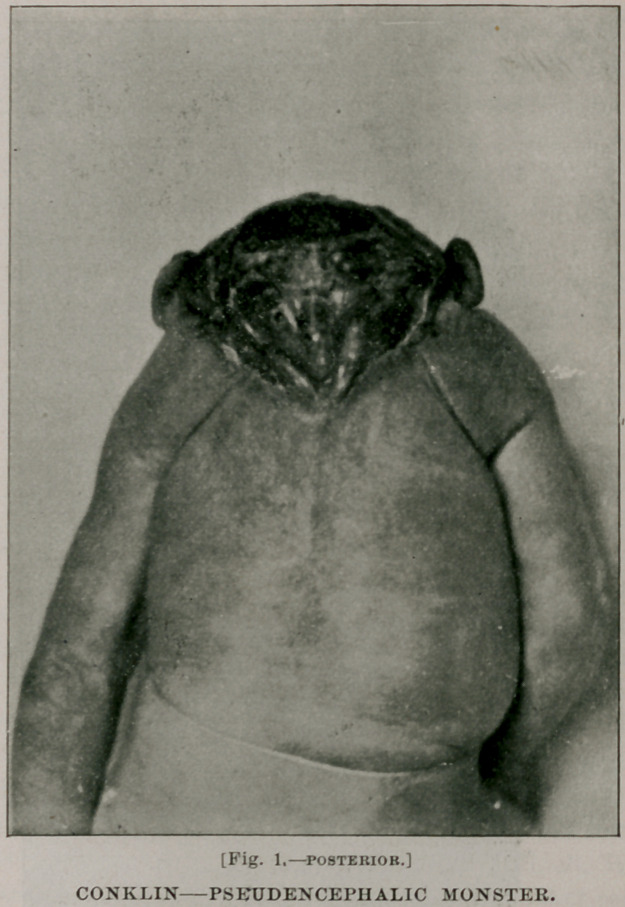


**Fig. 2. f2:**